# Brain Metastases in Patients With Soft-Tissue Sarcomas: Management and Survival—A SEER Population-Based Cohort Study

**DOI:** 10.5435/JAAOSGlobal-D-21-00219

**Published:** 2021-10-07

**Authors:** Marcos Roberto Gonzalez, Mayte Bryce-Alberti, Juan Alonso Leon-Abarca, Juan Pretell-Mazzini

**Affiliations:** From the Facultad de Medicina Alberto Hurtado, Universidad Peruana Cayetano Heredia, Lima, Perú (Gonzalez, Bryce-Alberti, and Leon-Abarca); the Instituto de Investigaciones de la Altura, Universidad Peruana Cayetano Heredia, Lima, Perú (Leon-Abarca); and the Department of Orthopedics, Division of Musculoskeletal Oncology, University of Miami Miller School of Medicine, Miami, FL (Pretell-Mazzini).

## Abstract

Brain metastases (BM) in soft-tissue sarcomas (STS) is associated with poor prognosis. This large population analysis presents (1) demographic and clinical variables of these patients, (2) potential risk factors, (3) impact of BM on overall survival, and (4) treatment strategies.

**Methods:**

Patients with STS and BM were identified from the Surveillance, Epidemiology, and End Results database. Demographic and clinical variables, as well as treatment modalities, were analyzed. Overall 5-year survival was calculated using the Kaplan-Meier method, and the survival difference was assessed using the log-rank test. A multivariate analysis was performed using the Cox proportional hazard regression to determine the risk factors.

**Results:**

Twenty-two patients (22/8,433) with STS presented BM at diagnosis. A multivariate analysis showed that women and American Indians/Alaska Natives had a greater risk of presenting BM. The most common histological subtype to metastasize to the brain was alveolar soft part sarcoma (4/22). In 54.5% of patients with BM, the tumor had also metastasized to the lung, although having synchronous bone, liver, and lung metastases showed the greatest increase in risk for presenting BM (odd ratio [OR] = 1,857.7, confidence interval [CI] 95%, 88.4 to 3,9046.6, *P* < 0.0001). Individually, bone metastasis increased the risk of presenting BM the most (OR = 205.0, CI 95%, 30.5 to 1,379.6, *P* < 0.0001). The mean survival of patients with BM was 10.22 months. The standard treatment approach included surgery, radiation therapy, and chemotherapy.

**Conclusion:**

BM in patients with STS represents an infrequent but lethal event. Women and American Indians/Alaska Natives are at a higher risk of presenting BM, as well as patients with synchronous metastases. Patients are mainly managed with systemic therapy.

Soft-tissue sarcomas (STS) are composed of approximately 50 different histologic subtypes, the lungs being the most common metastasis site.^1-3^ Advances in diagnosis and treatment include a multidisciplinary approach involving surgery, chemotherapy, radiation therapy (RT), and immunotherapy. Despite this, there is only a 55% overall survival at 5 years.^1-4^

Guidelines recommend that STS be staged with a chest CT to exclude pulmonary metastases before definite treatment.^1^ Nonetheless, other imaging strategies may be useful for STS that metastasize to different organs. Chan et al^5^ listed not screening for brain metastases (BM) as a limitation to their retrospective cohort study. Because all patients had neurological symptoms at the time of BM diagnosis, it was presumed that there was a lag in the detection of asymptomatic patients. Screening for BM may be more relevant in the presence of other systemic metastases because, in the aforementioned study, all patients had developed lung or bone metastases at a mean interval of 13.3 months before the development of BM. Because 3% of BM arise from sarcomas,^6,7^ authors have recommended considering routine screening for BM in patients with advanced disease; however, a lack of consensus still persists.^8,9^

Data concerning the optimal management of patients presenting with metastatic disease remains scarce. Traditionally, surgical resection with or without adjuvant RT represented the basis for treatment in patients with BM.^10^ Although this may be true for certain subtypes of STS,^5,7,11^ the development of combination therapies with monoclonal antibodies may be a game changer for specific patient populations.^12^

Owing to the overall lack of data regarding BM caused by STS, this large population study seeks to analyze (1) demographic and clinical characteristics of these patients, (2) risk factors that lead to BM, (3) the impact of BM on overall survival, and (4) the management options available.

## Methods

The Surveillance, Epidemiology, and End Results (SEER) database, sponsored by the National Cancer Institute, consists of 18 population-based cancer registries, accounting for approximately 28% of the US population. The study used the Incidence-SEER 18 Custom Data released in April 2019, which includes clinicopathological data such as demographics, tumor histology, tumor morphology, cancer stage at diagnosis, metastasis, and survival from 1975 to 2016. Data regarding treatment was added using the Radiation/Chemotherapy databases. The analysis performed was restricted to the period between 2010 and 2016 because no previous registry of data concerning metastasis to the bones, brain, liver, lung, and distant lymph nodes existed at the time of diagnosis.

We accessed the data using SEER*Stat, version 8.3.8, software. Search criteria were all patients presenting histologically confirmed STS, according to the “Site and Morphology-site recode ICD-O-3/WHO 2008,” diagnosed between 2010 and 2016. A total of 8,739 patients were identified. After excluding patients with unknown status on BM and/or incomplete follow-up data, and patients with double entries in the database, 8,433 patients (97% of total) remained.

Demographic variables included patient age, sex, race, marital status, insurance status, and region of origin. Clinical variables included tumor histologic subtype, tumor location and size, tumor grade, metastatic status—including synchronous lymph node, bone, liver, and lung involvement—and treatment strategies such as surgery, radiation, and chemotherapy.

A statistical analysis was performed using Stata software (StataCorp LLC). Demographic and clinical characteristics were analyzed using descriptive statistics. Overall, 1-, 2-, and 5-year survival was calculated using the Kaplan-Meier method. Survival difference was assessed using the log-rank test.

A multivariate analysis was performed using the Cox proportional hazard regression to determine notable risk factors for developing BM in patients with STS while controlling for additional variables. Variables analyzed in the multivariate analysis included demographic characteristics, primary location of the tumor, and other metastases (lung, liver, and bone). A *P* value ≤ 0.05 was considered statistically significant.

## Results

### Patient Characteristics

Of 8,433 patients with STS, only 22 patients (0.26%) had BM at the time of diagnosis. The mean age was 49.5 years, 59.1% (13/22) were women, and 59.1% (13/22) were Caucasian. In addition, 40.9% (9/22) were married or had a domestic partner, and 90.9% (20/22) were insured (private or Medicaid).

Approximately 86.4% (19/22) of the primary tumors were localized in the lower limb without laterality preference because 50% (11/22) of the tumors were located on each side. Patients with BM tended to have larger primary tumors with 27.3% (6/22) of them being >15 cm in their greatest dimension (T4 stage, AJCC 8th edition) (Table 1). Alveolar soft part sarcoma (18.2%, 4/22) was the most common histological subtype of STS in patients with BM, followed by angiosarcoma (13.6%, 3/22) and leiomyosarcoma (13.6%, 3/22) (Table 2).

**Table 1:** Demographic Characteristics of Patients with Soft-Tissue Sarcoma, With or Without Brain Metastases

	Total (n = 8,433)^a^	No Brain Met. (n = 8,411)^a^	Brain Met. (n = 22)	*P*-Value
Age	56.7 (20.5)^a^	56.7 (20.5)^a^	49.5 (21.4)^a^	0.1012
Follow-up time	30.0 (23.3)^a^	30.0 (23.3)^a^	10.2 (13.7)^a^	0.0001
Sex				
Male	4,703 (55.7%)	4,694 (44.2%)	9 (40.9%)	0.160
Female	3,730 (44.3%)	3,717 (55.8%)	13 (59.1%)	
Race				
Caucasian	6,661 (79.0%)	6,648 (79.0%)	13 (59.1%)	<0.0001
Black	954 (11.3%)	948 (11.3%)	6 (27.3%)	
American Indian/Alaska Native	57 (0.7%)	55 (0.7%)	2 (9.1%)	
Asian or Pacific Islander	666 (7.9%)	665 (7.9%)	1 (4.5%)	
Unknown	95 (1.1%)	95 (1.1%)	0 (0.0%)	
Marital status				
Single or never married	2,132 (25.3%)	2,124 (25.2%)	8 (36.4%)	
Separated, divorced, or widowed	1,470 (17.4%)	1,466 (17.4%)	4 (18.2%)	
Married or domestic partner	4,352 (51.6%)	4,343 (51.6%)	9 (40.9%)	
Unknown	479 (5.7%)	478 (5.7%)	1 (4.5%)	
Insurance				
Uninsured	287 (3.4%)	285 (3.4%)	2 (9.1%)	<0.0001
Medicaid	1,155 (13.7%)	1,143 (13.6%)	12 (54.5%)	
Insured	6,776 (80.4%)	6,768 (80.5%)	8 (36.4%)	
Unknown	215 (2.5%)	215 (2.6%)	0 (0.0%)	
Primary location				
Upper limb, shoulder	2,305 (27.3%)	2,302 (27.4%)	3 (13.6%)	0.149
Lower limb, hip	6,128 (72.7%)	6,109 (72.6%)	19 (86.4%)	
Laterality				
Right	4,103 (48.65%)	4,092 (48.65%)	11 (50.0%)	0.999
Left	4,320 (51.23%)	4,309 (51.23%)	11 (50.0%)	
Bilateral	1 (0.01%)	1 (0.01%)	0 (0.0%)	
Unknown	9 (0.11%)	9 (0.1%)	0 (0.0%)	
AJCC (8th Ed.)				
T1	2,658 (31.5%)	2,657 (31.6%)	1 (4.5%)	0.036
T2	2,471 (29.3%)	2,466 (29.3%)	5 (22.7%)	
T3	1,232 (14.6%)	1,228 (14.6%)	4 (18.2%)	
T4	1,298 (15.4%)	1,292 (15.4%)	6 (27.3%)	
Unknown	774 (9.2%)	768 (9.1%)	6 (27.3%)	
Grade				
I	717 (8.5%)	717 (8.5%)	0 (0.0%)	0.1
II	1,339 (15.9%)	1,339 (15.9%)	0 (0.0%)	
III	1,589 (18.8%)	1,584 (18.8%)	5 (22.7%)	
IV	2,705 (32.1%)	2,697 (32.1%)	8 (36.4%)	
Unknown	2,083 (24.7%)	2,074 (24.7%)	9 (40.9%)	

a Data displayed in age and follow-up time refer to the mean, whereas data in the parentheses refer to the SD. AJCC = American Joint Committee on Cancer.

**Table 2:** Most Common Histological Subtypes of Soft-Tissue Sarcoma That Cause Brain Metastases, Ranked According to Frequency

Histological Subtype	n	%	Age (Mean)	Survival (mo)
Alveolar soft part sarcoma	4	18.2	31	32.3
Angiosarcoma	3	13.6	52.7	4.0
Leiomyosarcoma	3	13.6	64.0	3.0
Sarcoma	3	13.6	68.7	0.0
Spindle cell sarcoma	2	9.1	53.0	9.0
Giant cell sarcoma	1	4.6	58	19.0
Small cell sarcoma	1	4.6	19	6.0
Malignant fibrous histiocytoma	1	4.6	59	1.0
Pleomorphic liposarcoma	1	4.6	63	5.0
Alveolar rhabdomyosarcoma	1	4.6	11	5.0
Clear cell sarcoma	1	4.6	58	4.0
Malignant peripheral nerve sheath tumor	1	4.6	35	17.0

### Risk Factors to Develop Brain Metastases

In more than half of the patients with BM, the tumor had also metastasized to the lung (54.5%, 12/22) and almost half of the patients presented bone metastases (40.9%, 9/22) (Table 3). A statistically significant difference existed in the rates of lymph node, lung, and bone involvement within comparison groups (patients with and without BM).

**Table 3:** Associated Metastases at the Time of Diagnosis in Patients With Extremity Soft-Tissue Sarcoma

	Total (n = 8,433)	No Brain Mets (n = 8,411)	Brain Mets (n = 22)	*P*-Value
Lymph node met.				
No	7,954 (94.3%)	7,939 (94.4%)	15 (68.2%)	<0.0001
Yes	287 (3.4%)	283 (3.4%)	4 (18.2%)	
Unknown	192 (2.3%)	189 (2.2%)	3 (13.6%)	
Lung met.				
No	7,800 (92.5%)	7,790 (92.6%)	10 (45.5%)	<0.0001
Yes	614 (7.3%)	602 (7.2%)	12 (54.5%)	
Unknown	19 (0.2%)	19 (0.2%)	0 (0.0%	
Liver met.				
No	8,354 (99.1%)	8,333 (99.1%)	21 (95.5%)	0.065
Yes	74 (0.9%)	73 (0.9%)	1 (4.5%)	
Unknown	5 (0.1%)	5 (0.1%)	0 (0.0%)	
Bone met.				
No	8,225 (97.5%)	8,212 (97.6%)	13 (59.1%)	<0.0001
Yes	201 (2.4%)	192 (2.3%)	9 (40.9%)	
Unknown	7 (0.1%)	7 (0.1%)	0 (0.0%)	

We conducted a crude and multivariate analysis to determine the risk factors for presenting BM. This analysis included all possible combinations of brain, lymph node, lung, liver, and bone involvement. The crude analysis showed that sex, race, insurance status, and other metastases (bone, liver, or lung) markedly modified the rates of BM (Table 4).

**Table 4:** Univariate (Crude) and Multivariate Regression (Adjusted) Analysis for Risk Factors for Brain Metastases in Patients With Extremity Soft-Tissue Sarcoma

	Crude				Adjusted (n = 6,687)			
	OR	95% CI		*P* Value	OR	95% CI		*P* Value
Sex								
Male (n = 8,433)	—	—	—	—	—	—	—	—
Female	1.82	0.78	4.27	0.166	4.4	1.22	15.87	0.023
Race								
Caucasian(n = 8,338)	—	—	—	—	—	—	—	—
Black	3.24	1.23	8.54	0.018	2.05	0.46	9.10	0.345
American Indian/Alaska Native	18.60	4.10	84.37	<0.0001	22.92	1.88	279.18	0.014
Asian or Pacific Islander	0.77	0.10	5.89	0.8	0.65	0.10	4.28	0.655
Age at diagnosis	0.98	0.97	1.003	0.092	1.02	0.99	1.05	0.238
Marital status								
Single or never married (n = 8,219)	—	—	—	—	—	—	—	—
Separated, divorced, or widowed	0.72	0.22	2.41	0.599	1.25	0.29	5.40	0.761
Married or domestic partner	0.55	0.21	1.43	0.22	1.37	0.32	5.83	0.672
Insurance								
Uninsured (n = 8,218)	—	—	—	—	—	—	—	—
Medicaid	1.50	0.33	6.72	0.599	5.98	1.45	24.70	0.014
Insured	0.17	0.04	0.80	0.025	—	—	—	—
Primary location								
Upper limb (n = 8,433)	—	—	—	—	—	—	—	—
Lower limb	2.39	0.71	8.07	0.162	1.03	0.18	5.76	0.972
Metastases								
None (n = 7,986)	—	—	—	—	—	—	—	—
Nodes	—	—	—	—	—	—	—	—
Lung	24.4	6.52	91.1	<0.0001	23.8	3.67	154.5	0.001
Lung + nodes	57.6	10.4	320.1	<0.0001	124.3	13.2	1,172.3	<0.0001
Liver	—	—	—	—	—	—	—	—
Liver + nodes	—	—	—	—	—	—	—	—
Liver + lung	—	—	—	—	—	—	—	—
Liver + lung + nodes	—	—	—	—	—	—	—	—
Bone	117.0	28.6	478.3	<0.0001	205.0	30.5	1,379.6	<0.0001
Bone + nodes	122.9	13.0	1,164.7	<0.0001	317.0	17.6	5,711.1	<0.0001
Bone + lung	35.4	3.89	322.5	0.002	85.2	5.45	1,330.7	0.002
Bone + lung + nodes	131.6	13.8	1,253.0	<0.0001	362.4	14.4	9,097.3	<0.0001
Bone + liver	—	—	—	—	—	—	—	—
Bone + liver + nodes	—	—	—	—	—	—	—	—
Bone + liver + lung	184.3	18.90	1,797.2	<0.0001	1,857.7	88.4	39,046.6	<0.0001
Bone + liver + lung + nodes	—	—	—	—	—	—	—	—
Tumor size, mm								
<50 (n = 7,660)	—	—	—	—	—	—	—	—
50-100	3.00	0.31	28.9	0.342	1.40	0.07	28.4	0.828
100-150	11.2	1.35	93.1	0.025	6.29	0.29	134.6	0.24
>150	10.1	1.22	84.3	0.032	4.56	0.20	102.4	0.339

Empty cells indicate subgroups without enough observations for comparisons.

The multivariate analysis showed that women had a 4.4 times greater risk of presenting BM (OR = 4.4, CI 95% 1.22 to 15.87, *P* = 0.023), and American Indians/Alaska Natives had a 22.92 times greater risk (OR = 22.92, CI 95% 1.88 to 279.18, *P* = 0.014). When analyzing additional metastases to the lymph node, lung, liver, and/or bone, having synchronous bone, liver, and lung metastases increased the risk of presenting BM the most (OR = 1,857.7, CI 95%, 88.4 to 39,046.6, *P* < 0.0001). However, when analyzed individually, having bone metastasis increased the risk of presenting BM the most (OR = 205.0, CI 95%, 30.5 to 1,379.6, *P* < 0.0001).

The histological grade of the tumor was not included in the multiple logistic model because it markedly reduced the small number of observations and could introduce bias.

### Impact of Brain Metastases on Overall Survival

**Figure 1:**
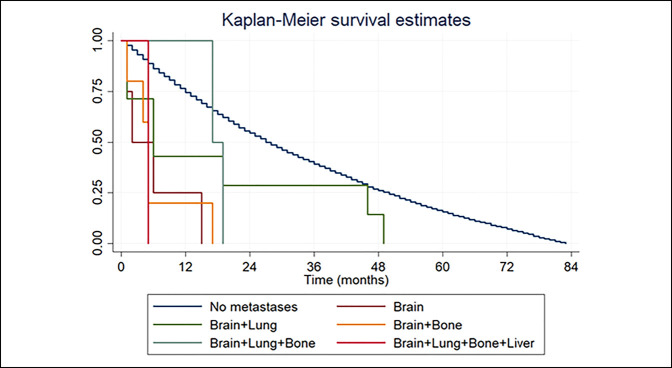
Chart showing the Kaplan-Meier survival estimates for patients with soft-tissue sarcoma according to the specific metastatic status (brain, lungs, bone, and/or liver metastases) based on the SEER database. SEER = Surveillance, Epidemiology, and End Results

The mean survival time of patients with BM was 10.22 months. Patients with isolated BM had the lowest survival time on average, living for 4.8 months after diagnosis (SD = 2.75) (Table 5). The Kaplan-Meier survival estimates showed that patients with brain and lung metastases lived the longest: 16 months (SD = 7.2) (Figure 1).

**Table 5:** Mean Survival (mo) in Patients With Brain Metastasis and Soft-Tissue Sarcoma, in Addition to Lungs, Bones, and/or Liver Metastasis

Associated metastasis	n	Mean	Median (IQR)	1-Year Survival		2-Year Survival	
				%	95% CI	%	95% CI
Only brain	5	4.8	2 (5)	20.0%	2.1-74.3%	—	—
Brain + lung	8	16	6 (31.5)	37.5%	11.5-73.5%	25.0%	5.7-64.9%
Brain + bone	5	6.4	5 (1)	20.0%	2.1-74.3%	—	—
Brain + lung + bone	3	12	17 (19)	66.7%	9.6-97.4%	—	—
Brain + lung + liver + bones	1	5	5 (0)	—	—	—	—

Data displayed refer to months. SD: standard deviation. IQR = interquartile range.

### Treatment Alternatives in Patients With Brain Metastases

Surgery to the primary site was performed in only 31.8% (7/22) of patients with BM, in comparison to 87.2% (7,334/8,411) of patients without BM (*P* < 0.0001) (Table 6). Because the SEER database does not specify where the surgery to the distant organ is performed, it only differentiates between surgery to a distant organ and surgery to distant lymph nodes; surgery to the brain could only be assessed in patients with BM and no other metastases. Of a total of five patients in this group, surgery to the brain was performed in one patient and surgery to the distant lymph nodes in one patient.

**Table 6:** Treatment Modalities Performed in Patients With Brain Metastasis and Soft-Tissue Sarcoma

	Total (n = 8,433)	No Brain Met. (n = 8,411)	Brain Met. (n = 22)	*P* Value
Qx to primary site				
No	1,016 (12.0%)	1,001 (11.9%)	15 (68.2%)	<0.0001
Tumor destruction	13 (0.2%)	13 (0.2%)	0 (0.0%)	
Resection	7,341 (87.1%)	7,334 (87.2%)	7 (31.8%)	
Qx to other sites				
No	8,251 (97.8)	8,235 (97.9%)	16 (72.7%)	<0.0001
Yes (No LN)	143 (1.7%)	138 (1.6%)	5 (22.7%)	
Yes (LN)	23 (0.3%)	22 (0.3%)	1 (4.5%)	
Radiation				
No	4,015 (47.6%)	4,007 (47.6%)	8 (36.4%)	0.290
Yes	4,418 (52.4%)	4,404 (52.4%)	14 (63.6%)	
Chemotherapy				
No	6,649 (78.8%)	6,638 (78.9%)	11 (50.0%)	0.001
Yes	1,784 (21.2%)	1,773 (21.1%)	11 (50.0%)	

LN = lymph node, Qx = surgery

We found no statistically significant difference in the overall use of RT in patients with and without BM. The SEER database does not differentiate between RT to the primary site and/or to the metastatic site; therefore, we can only assess the overall use of RT between study groups. However, our study shows that patients with BM were more likely to receive chemotherapy (50%, 11/22) than patients without (21.1%, 1,773/8,411) (*P* = 0.001).

## Discussion

### Patient Characteristics

The incidence of BM in STS is not clearly known, and the available reported prevalence is set at 1% to 6%.^13^ The prevalence obtained through this study falls out of this range at 0.26% (22/8,433); however, in contrast to this large patient population, most of the available literature is limited to small series and few small retrospective and prospective studies.^6,14^ Still, increasing use of new treatment options for systemic disease without proper intracranial management may lead to more cases being reported.^7^

The mean age of patients with BM in this study was 49.5 years. This value falls between the mean age reported by Salvati et al^7^ (33.1 years) and Chan et al^5^ (56.6 years). Both of these studies also demonstrated a male predominance unlike ours (59.1% of patients with BM were women). Further patient characteristics in available literature are not well described. With the vast number of patients selected for this population study, we were able to identify (Table 1) that 59% of patients with STS and BM were of Caucasian race. Additional findings included marital status (41% married or having a domestic partner) and insurance status (91% insured).

In accordance with our findings, studies show that most patients with BM had a lower limb primary tumor,^5,10^ with most being localized to the thigh.^10^ In addition, we encountered that patients with BM tended to have larger primary tumors with 27.3% being >15 cm in their greatest dimension (T4 stage). STS with BM are more likely high grade,^15^ and evidence shows that tumor size combined with higher grading is associated with poor outcomes.^16^

Our study identified alveolar soft-tissue sarcoma as the most common histological subtype of STS in patients with BM, followed by angiosarcoma and leiomyosarcoma. Alveolar soft part sarcomas have shown predisposition toward the brain with estimates of 15% to 30% in patients with stage IV disease,^6,17^ but with a longer mean survival when compared with the overall survival (32.3 versus 10.22 months) (Table 2). Unfortunately, most of the evidence concerning this subtype of STS derives from case reports and small series.^18^ In addition, a literature review by Shweikeh et al^10^ reported liposarcoma, rhabdomyosarcoma, malignant peripheral nerve sheath tumor, angiosarcoma, and alveolar soft part sarcoma as the most likely STS to metastasize to the brain.

### Risk Factors to Develop Brain Metastases

BM can be diagnosed at a median interval of 26 months from the diagnosis of the primary sarcoma and are usually preceded by pulmonary metastasis.^5^ In a retrospective analysis by Nakamura et al,^18^ 83% of the cohort developed lung metastasis as the first metastatic site. In addition, Salvati et al^7^ reported that six of nine patients with BM had synchronous lung metastases. This is concordant with our findings (Table 3): more than half of the patients with BM had a synchronous lung metastasis (54.5%). In addition to this trend, we also identified that 40.9% of patients with BM also had developed bone metastases. Overall, the group of patients presenting with BM presented a higher frequency of synchronous metastases compared with the group without BM. The findings by Chan et al^5^ showed that all patients with STS developed lung or bone metastases at a mean interval of 13.3 months before the development of BM. Similarly, a patient diagnosed with leiomyosarcoma of the right forearm was diagnosed with synchronous metastases to the brain, pancreas, and sacrum 8 years later.^11^ In our study, multiple metastases to bone, liver, and lung resulted in the highest risk of presenting BM (OR = 1,857.7, CI 95%, 88.4 to 39,046.6, *P* < 0.0001). On the other hand, when analyzed individually, having bone metastasis increased this risk the most (OR = 205.0, CI 95%, 30.5 to 1,379.6, *P* < 0.0001).

Currently, brain imaging and follow-up strategies have not been properly developed nor performed routinely after primary surgery in patients with STS.^1^ With these findings, and as Chan et al^5^ recognized in their study, not screening for BM, knowing that these may be asymptomatic and specially when the patient has already developed a previous distant metastasis may prevent physicians from making an early detection.

Other risk factors for developing BM in patients with STS were being women, with 4.4 times greater risk (OR = 4.4, CI 95% 1.22 to 15.87, *P* = 0.023) compared with men, and being American Indian/Alaska Native, with 22.92 times greater risk (OR = 22.92, CI 95% 1.88 to 279.18, *P* = 0.014).

### Impact of Brain Metastases on Overall Survival

Our findings showed that the mean survival time of this patient population was 10.22 months, which was similar to the findings by Salvati et al^7^ (10.5 months). Conversely, Chan et al^5^ reported a median interval from BM diagnosis to death of 1.7 months. One of the six patients with STS had a survival time after BM diagnosis of 47.1 months. This increased survival time was associated with metastases to the cerebellum, compared with the other patients who developed cerebral metastases. Survival time may also be influenced by the tumor histologic subtype because STS tend to be grouped together. For example, the mean overall survival after diagnosis of BM in patients with leiomyosarcoma is reported to range between 3 to 18 months^19^ and alveolar soft part sarcoma with 32.3 months as in our study.

Interestingly, in our study, patients with isolated BM had the lowest survival time on average (4.8 months after diagnosis; SD = 2.75) (Table 5). This could be attributed to the fact that synchronous metastases may be missed because of the lack of established surveillance protocols for metastases other than lung. For instance, within our population study, the Kaplan-Meier survival estimates showed that patients with brain and lung metastases lived the longest: 16 months (SD = 7.2) (Figure 1). Similarly, Salvati et al^7^ evidenced that patients with concurrent lung metastases did no worse than patients with no evidence of systemic disease.

### Treatment Alternatives in Patients With Brain Metastases

In response to conflicting evidence concerning the management approach for patients with BM, guidelines are nonspecific and consensus has not been reached.^1^ In general, addressing BM and other metastases requires a multidisciplinary approach.^15^ Our findings show that surgery to the primary site was performed in 31.8% (7/22) of patients with BM, in comparison to 87.2% (7,334/8,411) of patients without BM (*P* < 0.0001) (Table 6). Conversely, Chan et al^5^ reported that five of six patients with BM underwent wide local excision for the STS primary.

Traditionally, surgical resection with or without adjuvant RT represented the most popular treatment in patients with BM.^10^ This trend can be evidenced in our study showing surgical interventions to distant organs being more common in patients with BM (22.7% in patients with BM compared with 1.6% in patients without BM, *P* < 0.0001). Similarly, Salvati et al^7^ reported that all patients with BM underwent surgery for total removal of the lesion. Those in whom all known lesions were removed tended to live longer. Because resectability and prognosis are dependent on the number of metastases, it may prove convenient that compared with carcinomas, STS that metastasize to the brain, do so more often as a solitary BM.^15,20^

Chaigneau et al^15^ suggested that local treatments involving neurosurgical resection and RT improves the outcome of sarcoma patients with BM to a limited extent. Nevertheless, this cohort included both skeletal and nonskeletal sarcoma patients. The study also noted a positive correlation between medians outcome survival and the quality of response. Long survivors had a greater percentage of solitary BM lesions (52.9% versus 35.4%). In contrast, we found no statistically significant difference in the use of RT in patients with and without BM.

Chemotherapy is a treatment modality commonly used in advanced metastatic disease, and doxorubicin has been identified as the most effective of these agents for advanced STS.^21^ Following this trend, our study showed that when it came to chemotherapy, patients with BM were more likely to receive it than patients without BM (50 versus 21.1%, *P* = 0.001). However, although frequently used, chemotherapy may have a minor effect on BM.^22^

Interestingly, concerning alveolar soft part sarcoma, therapeutic options for advanced disease are limited because of the tumor's resistance to RT and chemotherapy.^23^ Xu et al^12^ reported a patient with stage IV alveolar soft part sarcoma in whom surgery was not recommended because of BM, bilateral lung metastases, and metastasis to the pancreas. Therefore, treatment involved a combination therapy of tyrosine kinase inhibitor apatinib and humanized programmed cell death 1 monoclonal antibody, camrelizumab. Partial remission was achieved with 46% and 69% remission from baseline after 3 months and 6 months, respectively. During the last follow-up in 2020 (10 months), the patient sustained partial remission. Although the treatment of metastatic alveolar soft part sarcoma with antiangiogenic agents such as apatinib, bevacizumab, and sunitinib has not effectively prevented recurrence, antibodies targeting programmed cell death 1 and its ligand may improve survival rates in these patients. More studies are necessary to explore this premise because better prognosis may have also been attributed to a cerebellar metastasis as was the case with the patients of Chan et al.^5^ With the arrival of new immunotherapeutic agents, prognosis may change in this patient population, especially in those whose tumor developed from fewer mutations.^12^

### Strengths and Limitations

The main strength of our study is the high number of patients included in our analysis, which allows us to assess extremely rare events such as BM in patients diagnosed with STS. However, several limitations exist because of the characteristics of the SEER database. First, our analysis was constrained to risk factors because of the nature of the database, which only included detected metastases at the time of diagnosis. In addition, therapeutic approaches were not detailed enough, and in patients with multiple metastases, the database did not specify whether RT and/or chemotherapy was applied to the primary tumor or to the metastases. Finally, important variables such as histologic grade had a percentage of values classified as “unknown” > 20%, which made the statistical analysis of such not feasible.

## Conclusion

BM in patients with STS represent an infrequent but lethal event. Women and American Indians/Alaska Natives are at a higher risk of presenting with BM. Having synchronous bone, liver, and lung metastases at the time of diagnosis has the greatest increase of risk to present BM. When analyzed individually, having bone metastasis increases the risk of presenting BM the most. Patients are mainly managed with systemic therapy.
